# Adjuvant and carrier protein-dependent T-cell priming promotes a robust antibody response against the *Plasmodium falciparum* Pfs25 vaccine candidate

**DOI:** 10.1038/srep40312

**Published:** 2017-01-16

**Authors:** Andrea J. Radtke, Charles F. Anderson, Nicolas Riteau, Kelly Rausch, Puthupparampil Scaria, Emily R. Kelnhofer, Randall F. Howard, Alan Sher, Ronald N. Germain, Patrick Duffy

**Affiliations:** 1 Laboratory of Systems Biology, NIAID/NIH, Bethesda, MD, USA; 2 Laboratory of Malaria Immunology and Vaccinology, NIAID/NIH, Rockville, MD, USA; 3 Laboratory of Parasitic Diseases, NIAID/NIH, Bethesda, MD, USA; 4 Infectious Disease Research Institute, Seattle, WA, USA.

## Abstract

Humoral immune responses have the potential to maintain protective antibody levels for years due to the immunoglobulin-secreting activity of long-lived plasma cells (LLPCs). However, many subunit vaccines under development fail to generate robust LLPC responses, and therefore a variety of strategies are being employed to overcome this limitation, including conjugation to carrier proteins and/or formulation with potent adjuvants. Pfs25, an antigen expressed on malaria zygotes and ookinetes, is a leading transmission blocking vaccine (TBV) candidate for *Plasmodium falciparum*. Currently, the conjugate vaccine Pfs25-EPA/Alhydrogel is in Phase 1 clinical trials in the USA and Africa. Thus far, it has proven to be safe and immunogenic, but it is expected that a more potent formulation will be required to establish antibody titers that persist for several malaria transmission seasons. We sought to determine the contribution of carrier determinants and adjuvants in promoting high-titer, long-lived antibody responses against Pfs25. We found that both adjuvants and carrier proteins influence the magnitude and capacity of Pfs25-specific humoral responses to remain above a protective level. Furthermore, a liposomal adjuvant with QS21 and a TLR4 agonist (GLA-LSQ) was especially effective at inducing T follicular helper (Tfh) and LLPC responses to Pfs25 when coupled to immunogenic carrier proteins.

Most vaccines mediate protection through the production of antibodies and are dependent on B cell differentiation in germinal centers (GCs). Within these structures, activated B cells compete for a limited number of Tfh cells, and upon receiving the appropriate signals, exit the GC reaction as memory B cells or plasma cells (PCs)^1^,^2^,^3^. Protection can be achieved through the generation of durable antibody responses or by high-titer antibody responses that rapidly decline but remain above a certain threshold over time. The first scenario may result from vaccine formulations that generate a LLPC response, whereas the second scenario may be the product of a large, but transient PC response. Both possibilities likely originate from distinct T-cell dependent antibody responses and are highly relevant for vaccine development against diseases of public health importance such as malaria.

While vaccines that target pre-erythrocytic and erythrocytic antigens are intended to confer protection and prevent clinical disease, vaccines that block parasite infectivity in the mosquito, termed TBVs, are aligned with the 2030 Strategic Goals of the Malaria Vaccine Technology Roadmap^4^. Accordingly, there has been considerable interest in the development of TBVs which induce antibodies that act primarily in the mosquito, rather than the mammalian host, to inhibit the parasite transmission cycle^5^. Pfs25, a leading TBV candidate antigen, is a 25 kDa *P. falciparum* sexual stage antigen expressed on zygotes and ookinetes in the mosquito following an infectious blood meal^6^,^7^. Although antibodies induced by vaccination with recombinant Pfs25 demonstrated functional activity in animals and humans^8^,^9^,^10^, long-lasting, protective titers were not achieved. Therefore, vaccine platforms employing other adjuvants and Pfs25 conjugates were investigated and shown to generate stable antibody responses with transmission-blocking activity^11^,^12^,^13^. Although these studies highlight the importance of immunogenic, nanoparticulate vaccines, a better understanding of the immune parameters underlying durability is needed. This is especially important because the protection mediated by TBVs may not be boosted by natural exposure and therefore would be exclusively dependent on vaccine-induced high-titer antibodies.

In the current work, we sought to improve the antigenicity and immunogenicity of Pfs25-based vaccines using clinically relevant carrier proteins and adjuvants. Protein carriers have traditionally been used with polysaccharide antigens to promote humoral responses by recruiting CD4^+^ T cells into the response^14^. Accordingly, we conjugated the Pfs25 protein to two different carrier proteins and examined the capacity of these conjugate vaccines to induce cellular responses, in particular Tfh cells. In addition to the benefit mediated by the carrier proteins, we evaluated the ability of various adjuvants to augment the immunogenicity of these conjugate vaccines. Beyond measuring the effects of these manipulations on the resulting antibody response, we examined how these changes in vaccine formulation influenced known correlates of humoral immunity using flow cytometry and confocal microscopy. We found that GLA-LSQ, a liposomal adjuvant formulation with a TLR4 agonist and QS21, profoundly impacted the magnitude of the Tfh and LLPC response against Pfs25, an effect that was further enhanced using Pfs25 conjugated to an antigenic carrier protein. Importantly, this adjuvant-dependent Tfh cell priming coincided with a large LLPC response and durable, functional antibody response. Together, our data provide insight into the immune responses elicited by unique vaccine formulations that enhance the quantity and quality of antibody responses against a malaria vaccine candidate.

## Results

### Adjuvants affect the magnitude and durability of antibody responses against Pfs25

**Figure 1:**
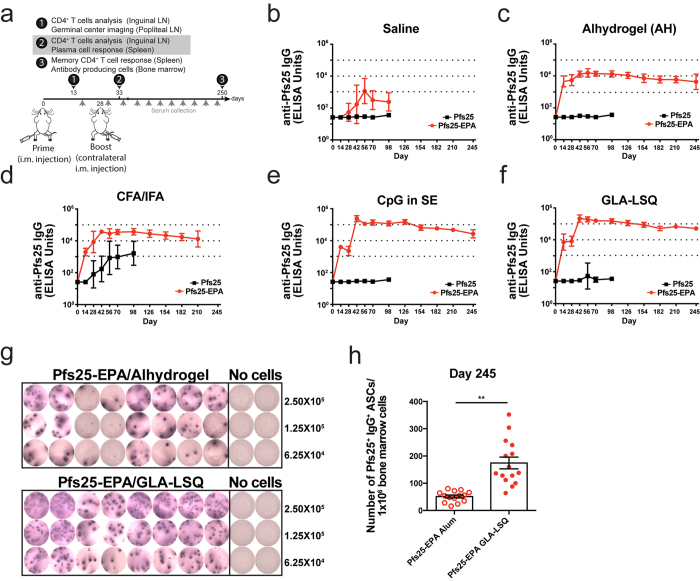
Adjuvants have a profound impact on the peak and magnitude of the long-lived antibody response. Mice were immunized i.m. with 1 μg Pfs25 alone (Pfs25) or Pfs25 conjugated to EPA (Pfs25-EPA) formulated in various adjuvants on day 0 and day 28. (**a**) Schema of immunization strategy. Mice were primed in the left leg and boosted, contralaterally, in the right leg. All mice were primed and boosted with the same antigen/adjuvant combination with the exception of the CFA/IFA group where mice were primed with CFA (s.c) and boosted with IFA (i.p.). (**b–f**) Anti-Pfs25 IgG ELISA titers collected at various time points after immunization with Pfs25-EPA and saline (**b**), Alhydrogel (**c**), CFA/IFA (**d**), CpG in SE (**e**), and GLA-LSQ (**f**). Shown is the geometric mean with 95% confidence interval from 5–10 mice per group. (**g**) Representative ELISPOTs showing ASC producing IgG antibodies reactive against Pfs25. Number of cells plated is noted to the right of the image. No cells refers to the negative control where no cells were plated. (**h**) Number of ASCs specific to Pfs25 in the bone marrow of mice on day 245 calculated via ELISpot. Shown is the mean ± SEM, n = 15–20 mice per group. (**P < 0.01; Mann-Whitney *U* test). Data are from one experiment and representative of 3 independent experiments.

A successful TBV will likely require high antibody titers that persist for several malaria transmission seasons. To this end, we investigated the relative contributions of adjuvants and carrier proteins to the magnitude and longevity of the antibody response against Pfs25. In these studies, non-conjugated Pfs25 (Pfs25) or Pfs25 conjugated to exoprotein A (Pfs25-EPA) was formulated in several distinct adjuvants or saline (Fig. 1a). Alhydrogel is an aluminum salt adjuvant^15^ currently used for Pfs25-EPA clinical trials (ClinicalTrials.gov Identifiers: NCT01434381 and NCT01867463). Although Freund’s adjuvant (CFA/IFA) has no clinical application, it was selected because it is a potent experimental adjuvant. In addition, we screened two proprietary adjuvants that have been formulated with other malaria vaccines and have potential clinical use: GLA-LSQ and CpG in SE^8^,^16^,^17^,^18^. GLA-LSQ is a liposome formulation containing the synthetic TLR4 ligand glucopyranosyl lipid adjuvant (GLA) and the saponin QS21, whereas CpG in SE contains a TLR9 ligand formulated in a stable emulsion. C57BL/6 mice received intramuscular (i.m.) immunizations on days 0 and 28 with 1 μg of Pfs25, or with Pfs25-EPA conjugates containing 1 μg of Pfs25, and anti-Pfs25 IgG responses were evaluated by enzyme-linked immunosorbent assays (ELISA) at the indicated time points. Mice vaccinated with non-conjugated Pfs25 did not produce appreciable antibody titers (Fig. 1b–f) except when the antigen was formulated with Freund’s adjuvant (Fig. 1d). However, the Pfs25-EPA conjugate in saline generated antibody titers above background, suggesting that Pfs25-EPA alone has some immune potentiating properties (Fig. 1b), as shown for other Pfs25 conjugates^12^. In contrast, adjuvanted vaccine formulations elicited large antibody responses and significant differences in peak titers were observed across all groups at day 42 (Fig. 1b–f). The Alhydrogel and CFA/IFA groups showed only a modest ~3-fold difference in peak titer, whereas the GLA-LSQ and CpG in SE groups induced peak titers that were more than 5-fold higher than the Alhydrogel and CFA/IFA groups (Table 1).

To address adjuvant contributions to durability, anti-Pfs25 antibody titers were monitored over 245 days and the rate of IgG decay in anti-Pfs25 titers was measured between days 126 and 245. This time frame was selected because the resulting antibody response would likely be sustained by LLPCs, a population of antibody-secreting cells (ASCs) shown to persist for 90 days after booster immunization without turnover^19^. We observed considerable differences in the antibody half-lives across all adjuvant groups with the CpG in SE formulation generating the shortest half-life, ~56 days, and Freund’s adjuvant generating the longest half-life, ~104 days (Table 1). Given the importance of LLPCs in maintaining durable antibody responses^19^,^20^,^21^,^22^, mice were sacrificed at the end of the study (>200 days) and the anti-Pfs25 IgG LLPC response in the bone marrow was measured by Enzyme-Linked ImmunoSpot (ELISpot) (Fig. 1g,h and Figure S1a). Consistent with the ELISA data (Fig. 1b–f), the Pfs25-EPA/GLA-LSQ vaccine formulation had the greatest number of antigen-specific antibody secreting cells (ASC) in the bone marrow at day 245 (Fig. 1h and Table 1). Furthermore, no ASCs were observed in the non-adjuvanted saline group and the CFA/IFA, CpG in SE, and Alhydrogel groups had ~38%, 55%, and 71% fewer LLPCs than the GLA-LSQ group, respectively (Figs 1h and S1a). In summary, antigen-specific antibody titers and ASC numbers coincided at later time points (>200 days); however, differences in antibody decay rates were observed between groups. This lack of uniformity cannot be attributed to a simple model where adjuvants induce different numbers of LLPCs and therefore suggests distinct adjuvant-controlled LLPC lifespans. While these results highlight the contribution of four distinct adjuvants to antibody magnitude and durability (Fig. 1 and Table 1), subsequent experiments focused on Alhydrogel and GLA-LSQ, two clinically relevant adjuvants that generated significant differences in the Pfs25-specific humoral response. Furthermore, the selection of GLA-LSQ as the main comparator to Alhydrogel is supported by the fact that another liposome-based adjuvant system containing a TLR4 agonist and QS21 has been recently approved for clinical use^23^ and shown to promote durable adaptive immune responses in human vaccine trials^24^,^25^.

### Antigenic carrier proteins are required for an optimal adjuvant-induced antibody response

**Figure 2:**
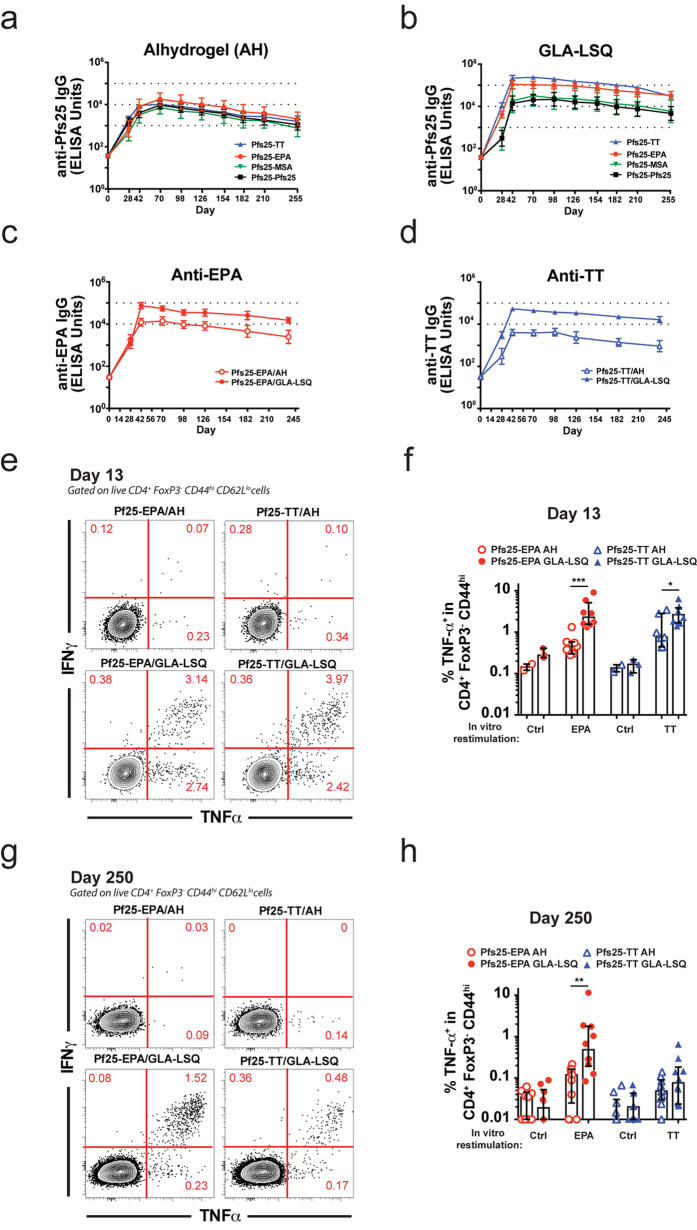
Humoral and cellular responses to carrier proteins. Mice were immunized i.m. with 1 μg Pfs25 conjugated to TT (Pfs25-TT), EPA (Pfs25-EPA), mouse serum albumin (Pfs25-MSA), or self-conjugated (Pfs25-Pfs25) formulated in Alhydrogel (AH) or GLA-LSQ on day 0 and day 28. (**a,b**) Anti-Pfs25 IgG ELISA titers were collected at various time points after immunization with conjugate vaccines in Alhydrogel (**a**) or GLA-LSQ (**b**). (**c**) Anti-EPA IgG ELISA titers collected at various time points after immunization. (**d**) Anti-TT IgG ELISA titers collected at various time points after immunization. Shown is the geometric mean with 95% confidence interval from 5–10 mice per group. Inguinal LNs (**e,f**) and spleens (**g,h**) were harvested at the indicated time points and processed for flow cytometry. Lymphocytes were left untreated (Ctrl), restimulated with EPA protein (10 μg/ml) or with TT protein (20 μg/ml) for 2 hours and then brefeldin A was added for 5 additional hours. Representative flow cytometry plots showing the frequency of TNF-α^+^ and/or IFN-γ^+^ within the activated CD44^hi^Foxp3^−^CD4^+^ T cell population 13 (**e**) and 250 (**g**) days after immunization. Frequency of TNF-α producers within the activated CD44^hi^Foxp3^−^CD4^+^ T cell population 13 (**f**) and 250 (**h**) days after immunization. Shown is the mean ± SEM, n = 5–10 mice per group. (*P < 0.05, **P < 0.01, ***P < 0.001; Mann-Whitney *U* test). All data presented here are representative of 3 independent experiments.

Our previous experiments (Fig. 1) and others^11^,^12^,^13^,^26^ demonstrated that a conjugated form of Pfs25 is required to increase its capacity to induce humoral responses. We hypothesized that the carrier protein used for conjugation imparts antigenic properties and therefore designed experiments to dissect the role of carrier proteins in eliciting long-lived antibody responses. We conjugated Pfs25 to itself (Pfs25-Pfs25) to create a nanoparticle. Additionally, we conjugated Pfs25 to mouse serum albumin (Pfs25-MSA), generating a particulate antigen without added non-self T or B cell epitopes, and to tetanus toxoid (Pfs25-TT), a foreign carrier protein used in commercial vaccines against polysaccharide antigens^27^. Each of these chemical conjugates formed a nanoparticle, consistent with Pfs25-EPA, and therefore provided a valid comparison of different carriers using the same platform. Mice received intramuscular immunizations with Pfs25-Pfs25, Pfs25-MSA, Pfs25-TT, or Pfs25-EPA formulated with Alhydrogel or GLA-LSQ on days 0 and 28 and antibody responses were monitored over time (Fig. 2a and b). Within the Alhydrogel groups, we found modest differences in the anti-Pfs25 IgG antibody response between the Pfs25-EPA and Pfs25-TT groups as compared to the Pfs25-Pfs25 and Pfs25-MSA groups both at peak titer (day 42) and at the termination of the study (Fig. 2a and Table S1). In contrast, a significant fold-increase in antibody titers was observed for Pfs25-EPA and Pfs25-TT formulated with GLA-LSQ as compared to Pfs25-Pfs25 and Pfs25-MSA (Fig. 2b and Table S1). Interestingly, there was a ~2-fold difference in the antibody response at peak titer between the Pfs25-EPA and Pfs25-TT conjugates formulated with GLA-LSQ (Fig. 2b). This prompted us to investigate quantitative and qualitative differences in the LLPC response using flow cytometry (Figure S1b–e). The frequency (Figure S1b) and total number (Figure S1c) of Pfs25^+^IgG^+^ specific PCs in the bone marrow were substantially higher in mice immunized with Pfs25-EPA or Pfs25-TT formulated with GLA-LSQ as compared to Alhydrogel. However, there were no clear differences in the mean fluorescence intensities of intracellular staining for Blimp-1 and IRF4, two key factors required for PC development^1^, nor following staining for IgG or cell binding to Pfs25, our surrogates for antibody quantity and affinity, respectively, in the LLPCs generated under these conditions (Figure S1d and S1e).

To further investigate the antigenic properties of these carrier proteins, we immunized mice i.m. with the two conjugate vaccines, Pfs25-EPA and Pfs25-TT, in Alhydrogel and GLA-LSQ on day 0 and 28 and measured antibody titers against EPA and TT over time (Fig. 2c and d, Table S2). Similar to anti-Pfs25 IgG titers, we observed an adjuvant dependent effect on anti-EPA and anti-TT IgG titers, with GLA-LSQ inducing a greater than 6-fold increase in antibody titers at day 42 (peak) and day 236 (end-point) for both carrier proteins (Fig. 2c and d, Table S2). In addition to humoral immune responses, we also examined carrier-specific cytokine production by effector and systemic memory CD4^+^ T cells in the inguinal LN (day 13) and in the spleen (day 250), respectively. CD4^+^ T cells produced abundant levels of TNF-α after *in vitro* stimulation with TT and EPA 13 and 250 days after immunization with Pfs25-EPA and Pfs25-TT (Fig. 2e–h). However, cytokine production was not detected after stimulation with Pfs25 (data not shown). We hypothesized that the poor antigenicity of Pfs25 may be due to its lack of CD4^+^ T cell epitopes and queried the Pfs25, EPA, and TT proteins with a custom C++ script developed by Marc Jenkins’ laboratory to identify I-A^b^ binding peptides^28^. Indeed, EPA and TT possessed several CD4^+^ T cell epitopes, whereas Pfs25 had only one peptide above the prediction threshold (Table S3). These results suggest that the Pfs25-EPA and Pfs25-TT conjugate vaccines contain CD4^+^ T cell epitopes that, when formulated with a strong adjuvant like GLA-LSQ, enhance humoral immunity compensating for the deficiency in T epitopes in the malaria antigen itself.

### GLA-LSQ adjuvant induces robust Tfh cell, plasmablast, and functional antibody responses

**Figure 3:**
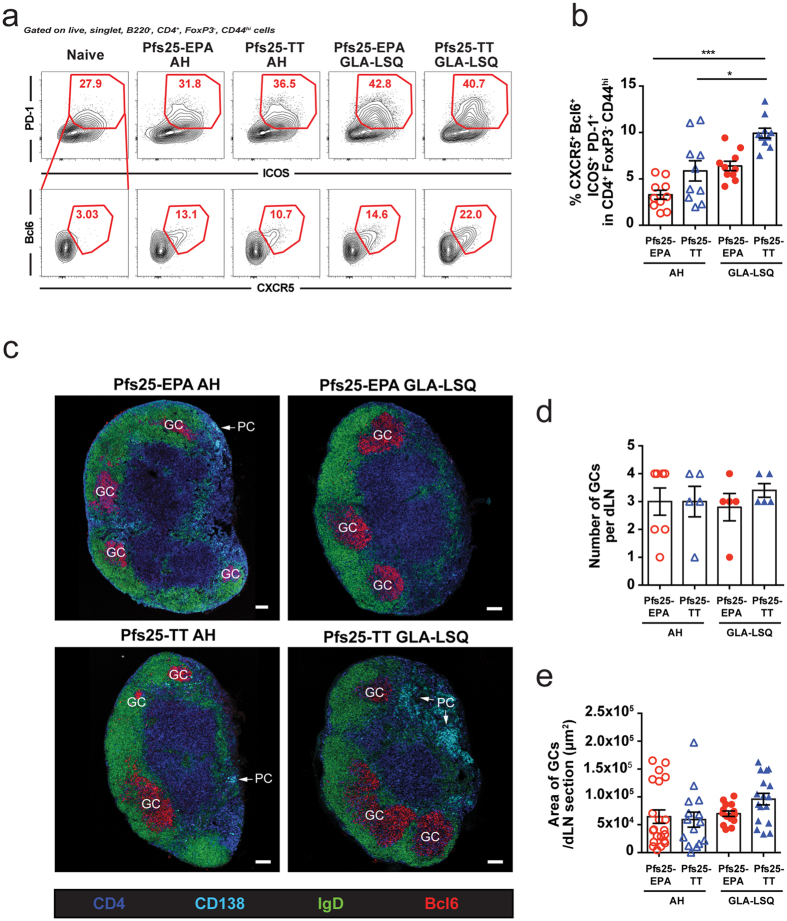
Evaluation of early Tfh cell and GC responses in the dLNs (Day 13). Mice were immunized i.m. with 1 μg Pfs25-EPA or Pfs25-TT in Alhydrogel or GLA-LSQ. dLNs were harvested on day 13 and examined by flow cytometry (inguinal LN) or confocal microscopy (popliteal LN). (**a**) Representative flow cytometry plots showing, within the activated CD44^hi^Foxp3^−^CD4^+^ T cell population, the frequency of ICOS^+^PD-1^+^ cells (top row) and within this population the frequency of Bcl6^+^CXCR5^+^ cells (bottom row). (**b**) Frequency of ICOS^+^PD-1^+^CXCR5^+^Bcl6^+^ Tfh cells within the CD44^hi^Foxp3^−^CD4^+^ T cell population. (**c–e**) Popliteal lymph nodes (dLN) were harvested 13 days post-immunization, stained with the indicated antibodies, and examined by confocal microscopy. (**c**) Representative dLN sections from 5–6 dLNs per mouse with germinal centers denoted by GC and plasma cells denoted by PC. Colors of the word labels correspond to the colors of the stains here and throughout. Scale bar corresponds to 100 μm. (**d**) Number of GC reactions per dLN. Each symbol represents a dLN. (**e**) Area of GC reactions per dLN section. Area was calculated from the widest part of each GC. Each symbol represents one GC per 5–6 whole dLNs. Shown is the mean ± SEM. (*P < 0.05, ***P < 0.001; One-way ANOVA with Tukey post-test). Data are pooled from 2 similar experiments.

Given the cellular response elicited by the carrier proteins, we decided to focus on their capacity to induce Tfh cells, a subset of CD4^+^ T cells required for germinal center (GC) B cell differentiation into LLPCs and memory B cells^3^. As described above, mice received i.m. immunizations with Pfs25-EPA or Pfs25-TT formulated in Alhydrogel or GLA-LSQ. The inguinal lymph nodes (dLNs) were harvested at the peak of the Tfh cell response (day 13), and the percentage of Tfh cells, identified as CXCR5^+^Bcl6^+^ICOS^+^PD-1^+^ cells within the CD4^+^Foxp3^−^CD44^hi^ population, was evaluated by flow cytometry (Fig. 3a). At this time point, the frequency of Tfh cells was significantly higher following immunization with Pfs25-TT in GLA-LSQ as compared to Pfs25-TT and Pfs25-EPA in Alhydrogel (Fig. 3b). In parallel, we also examined the GC response in popliteal LNs by confocal microscopy using antibodies directed against CD4^+^ T cells, naïve B cells (IgD), PCs (CD138), and Bcl6, the master regulator of Tfh^29^,^30^,^31^ and GC B cells^32^,^33^ (Fig. 3c). Although the average number of GC reactions was relatively stable across all groups examined (Fig. 3d), the size of GCs was highly variable (Fig. 3e). In particular, the smallest and largest GCs were elicited by Alhydrogel, whereas homogenously medium to large GCs were present in the GLA-LSQ groups (Fig. 3e).

**Figure 4:**
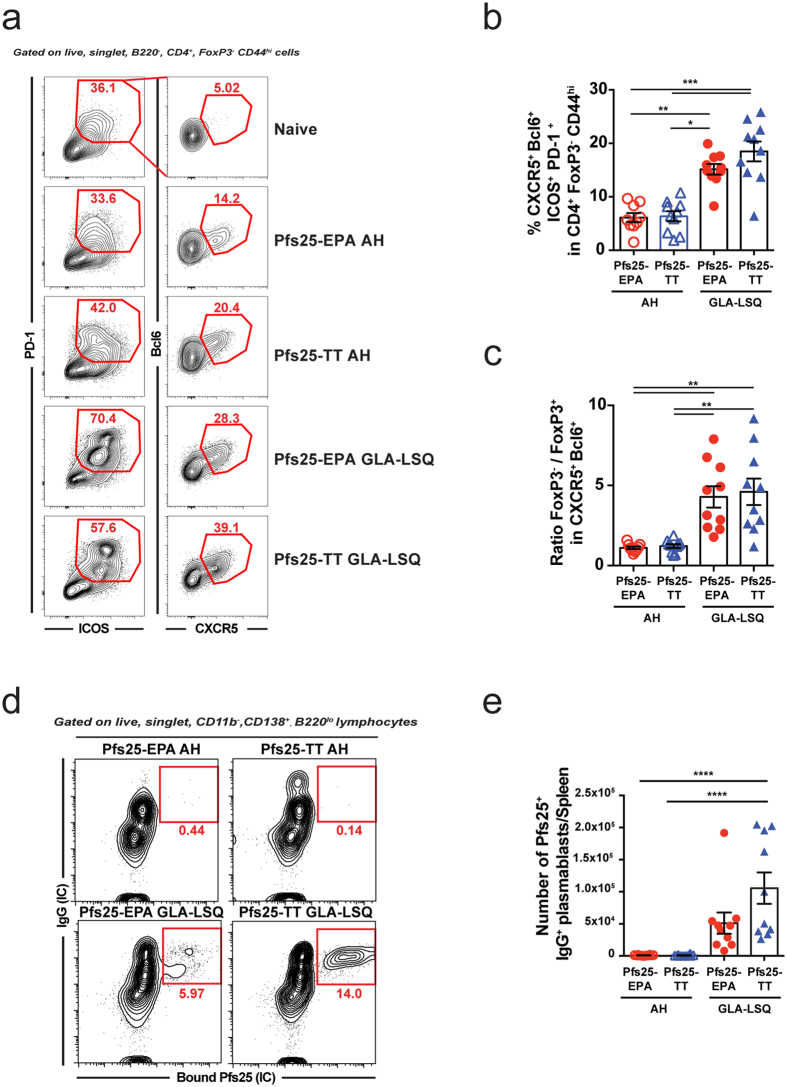
GLA-LSQ adjuvant promotes rapid expansion of Tfh cells and plasmablasts after boost immunization (Day 33). Mice were immunized i.m. with 1 μg Pfs25-EPA or Pfs25-TT in Alhydrogel or GLA-LSQ on day 0 and day 28. Spleens and inguinal LNs were harvested on day 33 post-immunization and processed for flow cytometry. (**a**) Representative flow cytometry plots showing, within the activated CD44^hi^Foxp3^−^CD4^+^ T cell population, the frequency of ICOS^+^PD-1^+^ cells (left column) and within this population the frequency of Bcl6^+^CXCR5^+^ cells (right column) in the inguinal LN. (**b**) Histogram showing the frequency of ICOS^+^PD-1^+^CXCR5^+^Bcl6^+^ Tfh cells within the CD44^hi^Foxp3^−^CD4^+^ T cell population in the inguinal LN. (**c**) Histogram showing the ratio of Foxp3^−^ (Tfh cells) versus Foxp3^+^ (Tfr cells) within the CXCR5^+^Bcl6^+^ CD4^+^ T cell population in the inguinal LN. (**d**) Expansion of Pfs25^+^IgG^+^ specific plasmablasts in the spleens of immunized mice on day 33. IC denotes intracellular staining with fluorescent Pfs25 or with an anti-IgG antibody. (**e**) Number of Pfs25^+^IgG^+^ splenic plasmablasts on day 33 calculated via flow cytometry. Shown is the mean ± SEM, n = 10 mice per group. Data are pooled from 2 similar experiments. (*P < 0.05, **P < 0.01, ***P < 0.001, ****P < 0.0001; One-way ANOVAs with Tukey post-tests).

While both adjuvants induced robust Tfh cell and GC responses after one immunizing dose, vaccine boosts are known to alter the output of secondary GCs and promote LLPC differentiation^34^. For this reason, mice received a homologous vaccine boost on day 28 and lymphoid organs were harvested five days later on day 33. At this time point, significantly higher frequencies of Tfh cells were observed in the inguinal LNs of mice immunized with GLA-LSQ when compared to mice receiving Alhydrogel (Fig. 4a and b). In addition, we evaluated the ratio of Tfh cells to T follicular regulatory (Tfr) cells (Figure S2a and S2b), a subset of cells that suppress Tfh and GC B cell responses^35^. Interestingly, mice immunized with the GLA-LSQ formulation had a higher Tfh to Tfr cell ratio than their Alhydrogel counterparts (Fig. 4c). Accordingly, we observed a significant expansion of antigen-specific plasmablasts in the spleen following two immunizations with the GLA-LSQ adjuvant and a near absence of these cells in both Alhydrogel groups (Fig. 4d and e). Together, these results suggest that the GLA-LSQ formulation induces qualitative differences in T helper differentiation, a higher Tfh:Tfr cell ratio, that may influence GC output and memory development after secondary immunization. Despite a ~2-fold increase in the number of splenic plasmablasts generated by Pfs25-TT versus Pfs25-EPA in the GLA-LSQ formulation (Fig. 4d and e), no differences in Blimp1, IRF4, and intracellular IgG levels were found between these two groups (Figure S3a and b). However, the Pfs25-TT conjugate vaccine did induce plasmablasts that bound more fluorescent Pfs25 antigen (Figure S3b).

**Figure 5:**
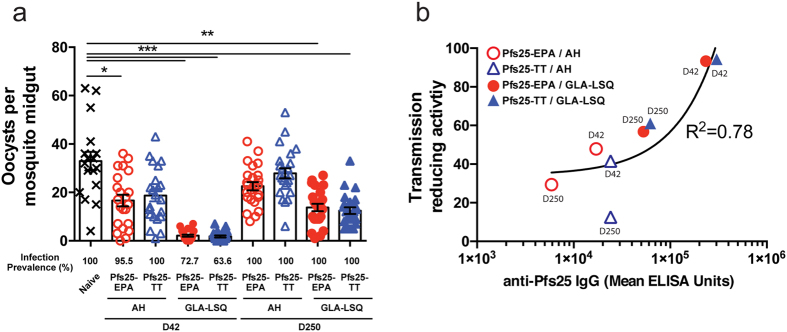
Adjuvant-dependent induction of functional antibodies with transmission-reducing activity. Mice were immunized i.m. with 1 μg Pfs25-EPA or Pfs25-TT in Alhydrogel or GLA-LSQ on day 0 and day 28. Sera were collected on day 42 or 250 post-immunization, pooled from 10 mice per group, and diluted (1:8) with naïve sera. Diluted sera and *P. falciparum* NF54 gametocytes were fed to *Anopheles stephensi* mosquitoes and the oocyst numbers in mosquito midguts were counted as an indication of infectivity. (**a**) Number of oocysts per mosquito as determined by membrane feeding assays (MFA). Pre-immune sera are from naïve controls. Shown is the mean ± SEM, n = 22–24 dissected mosquitoes per group. Infection prevalence (number of mosquitoes infected/number of mosquitoes dissected) is expressed as a percentage beneath the x-axis. (**b**) Comparison of transmission-reducing activity of sera relative to ELISA titers. Data shown are mean ELISA titers versus percentage of oocyst reduction as compared to control. The black line shows the nonlinear regression fit of the data. Data are from 1 experiment and are representative of 2 experiments conducted with 2 separate feeds. (*P < 0.05, **P < 0.01, ***P < 0.001; One-way ANOVA with Tukey post-tests).

Importantly, the increased expansion of Tfh cells and plasmablasts observed after secondary immunization with the GLA-LSQ adjuvant formulation corresponded with higher peak (day 42) and long-term (day 250) anti-Pfs25 IgG titers. Therefore, we next assessed how these adjuvant-dependent differences in antibody titer and duration impacted biological activity using a membrane feeding assay (MFA). To assess the capacity of these antibodies to neutralize parasite development in the mosquito host, sera from day 42 (peak) or day 250 (termination of study) were pooled from mice immunized with Pfs25-EPA or Pfs25-TT formulated in Alhydrogel or GLA-LSQ. In all the immunization groups examined, oocyst numbers were reduced in mosquitoes fed on membranes with sera from day 42 (Fig. 5a and b). However, sera from the GLA-LSQ groups significantly inhibited parasite development in the mosquito at day 42 and, most importantly, at day 250 (Fig. 5a and b). These findings demonstrate the potential of the GLA-LSQ adjuvant to maintain titers above an efficacious threshold for a longer period of time than Alhydrogel. In summary, the GLA-LSQ adjuvant promotes a durable and functional antibody response that is preceded by increased expansion of Tfh cells and a greater plasmablast burst.

## Discussion

Nearly all licensed vaccines protect the host through antibodies rather than cell-mediated responses. Therefore, the primary goal of this study was to identify aspects of T-cell dependent antibody responses that influence the persistence of high antibody levels after vaccination, with a specific focus on the choice of adjuvants and carrier proteins in vaccine formulations against Pfs25, a leading TBV candidate antigen. Pfs25-based vaccines have been shown to induce antibodies with transmission-blocking activity; however, sustained antibody titers are likely required to maintain this biological activity above a protective threshold. This is especially important as the blocking efficacy of TBVs was previously shown to depend more on the magnitude of the antibody response rather than the isotype generated^8^. An additional concern is the poor antigenicity of Pfs25, a small protein that, in many ways, behaves like a hapten. Several methods have been used to overcome this limitation including: its conjugation to carrier proteins, antigen multimerization, and formulation with potent adjuvants^8^,^10^,^11^,^12^,^36^. While a Pfs25-EPA/Alhydrogel vaccine exhibited an excellent safety profile and demonstrated transmission-blocking activity in humans, antibody levels rapidly declined, indicating that a more immunogenic vaccine is likely needed to interrupt malaria transmission^37^. In this study, we conjugated Pfs25 to two carrier proteins that are either licensed, TT^27^, or in Phase I clinical trials, EPA, in humans^37^. Both carrier proteins generated humoral and cellular responses that were significantly above background and persisted at substantial levels until the termination of the study. Furthermore, we demonstrated that the EPA and TT carrier proteins could generate Tfh cells and identified putative I-A^b^-binding epitopes in these carrier proteins. In contrast, Pfs25-specific CD4^+^ T cell responses were not detected after *ex vivo* stimulation, consistent with the paucity of epitopes predicted by the I-A^b^ binding algorithm. These findings clearly demonstrate that the antigenicity of conjugate vaccines matters and thus, a successful TBV will undoubtedly require inclusion of carrier proteins with peptides that bind HLA “supertypes” or the most common HLA types in humans^38^.

In addition to possessing B and T cell epitopes, the immunogenicity of vaccine formulations significantly contributes to the strength and quality of the immune response. Traditionally, adjuvants have been used to promote antigen uptake and display, induce inflammatory cascades in antigen presenting cells, and influence vaccine biodistribution^39^,^40^. However, a careful balance between enhanced potency and reactogenicity must be maintained to avoid adverse events, e.g., local tenderness and more serious systemic reactions (erythema nodosum), as reported in a Phase 1 clinical trial of two malaria TBV candidates, Pfs25 and Pvs25, formulated with the Montanide ISA 51 adjuvant^10^. In the present study, we used a variety of clinically relevant and experimental adjuvants, with the view that the latter may foster desirable responses and provide guidance into the development of acceptable alternatives for human use. We observed a profound adjuvant-dependent effect on both the magnitude and durability of the antibody response after vaccination with Pfs25-EPA. Interestingly, the highest antibody titers were observed in mice vaccinated with adjuvant formulations containing TLR ligands—CpG in SE (TLR9) and GLA-LSQ (TLR4)—an effect that is likely due to the direct engagement of these TLRs expressed by dendritic cells and B cells. However, antibody titers elicited by the CpG in SE adjuvant declined rapidly and had a much shorter antibody half-life than Alhydrogel and GLA-LSQ. One explanation for these differences in antibody duration may be the capacity of these adjuvants to retain antigen for long periods of time at the injection site^39^,^40^ or as persistent GCs, as previously shown for nanoparticles containing TLR4 and TLR7 ligands^41^. Alternatively, the physical characteristics (liposome versus emulsion), immune potentiating properties (type and quantity of TLR agonists), and ability to engage multiple pattern recognition receptors in distinct antigen-presenting cells (APCs) may influence the quality and longevity of the response. For example, the GLA-LSQ adjuvant has been shown to activate TLR4-dependent pathways via GLA^42^ and the NLRP3-inflammasome via QS21^43^. Due to species-specific differences in TLR expression, function, and ligand sensitivity, caution must be exercised before extrapolating results obtained with pre-clinical animal models to humans. For example, TLR4 is expressed by murine pDCs and naïve B cells, but is noticeably absent from their human counterparts^44^. Furthermore, while TLR9 is expressed in B-lymphocytes and pDCs in both species, additional DC subsets also express TLR9 in mice but not in humans^45^.

In additional studies examining the capacity of two clinically relevant adjuvants to augment antibody responses elicited by Pfs25-EPA and Pfs25-TT, we found that both of these conjugate vaccines generated 10-fold higher antibody titers when formulated with the GLA-LSQ adjuvant as compared to Alhydrogel. Furthermore, polyfunctional carrier-specific CD4^+^ T cell responses (i.e., cells that produce more than one cytokine) were induced by the GLA-LSQ formulation and sustained for 250 days. These adjuvant-dependent differences in humoral and cellular responses corresponded with greater Tfh cell and plasmablast responses after two immunizations with the GLA-LSQ adjuvant and are consistent with previous work establishing a linear relationship between the magnitude of Tfh and GC B cell responses^46^,^47^. In addition to affecting the extent of the Tfh cell response, certain adjuvants strongly promote CD4^+^ T cells to adopt a Tfr phenotype^48^ and, indeed, we observed a ~1:1 ratio of Tfh:Tfr with Alhydrogel versus a ~4:1 ratio with the GLA-LSQ adjuvant formulation. Ongoing studies include the identification of adjuvants that favor Tfh development over Tfr as well as the creation of TBV-specific tetramers to relate Tfh quality and quantity to vaccine outcome.

Although transmission-reducing activity was observed at the termination of the study in the GLA-LSQ immunization groups (>250 days), antibody titers declined in all the groups examined. This finding likely reflects an inherent deficiency of subunit vaccines to induce long-lasting antibody responses as compared to several licensed vaccines that result in viral infection^49^. However, the increased potency and safety profile of liposomal-based adjuvants provides an attractive strategy to induce the exceptionally high antibody titers required for an effective TBV. Therefore, we anticipate that vaccines employing antigenic carrier proteins and immunogenic adjuvants can enhance TBV-mediated protection through the generation of a durable antibody response sustained by LLPCs or by inducing a large, transient PC response that maintains protective titers above a minimal threshold over an extended time frame.

## Methods

### Animals and Immunizations

All animal procedures were performed according to protocols approved by the NIAID and NIH Animal Care and Use Committee. 5–8 week old naïve, female C57BL/6 mice were purchased from Jackson Laboratories (Bar Harbor, ME) or Taconic Laboratories (Hudson, NY) and maintained at a facility at the NIH. All procedures were performed in accordance with an animal study protocol approved by the NIAID Animal Care and Use Committee. Immunizations were performed by intramuscular (i.m.) injection (GLA-LSQ, CpG in SE, Alhydrogel, or saline) in the rear thigh, or subcutaneous (s.c.) and intraperitoneal (i.p) (CFA/IFA) in a volume of 50 μL containing 1 μg of Pfs25 for the various conjugate vaccines. For i.m. injections, the prime and boost doses were performed in alternating limbs (See Fig. 1a). Please see Supplementary Information for additional details on the proteins, conjugates, and adjuvants used for these studies.

### Measurement of Humoral Responses

Antigen-specific antibody responses were measured by ELISAs and B cell ELISpots. Please see Supplementary Information for additional details. Antibody half-lives were calculated using antibody titer data from day 126 and day >200 using the following formula: 
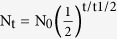
 where N_0_ is the antibody titer at day 126, N_t_ is the antibody titer at day >200, and t is the time (days) elapsed between N_t_ and N_0_ (t = 200–126 = 74 days).

### Flow Cytometry

Single cell suspensions were prepared from the spleen and inguinal dLNs by mashing organs through 100 μm cell strainers. To collect bone marrow cells, femurs and tibias were cleaned of all connective tissue using a scalpel, the epiphyses of the bone were cut with a scissors, and the bone was flushed using a 30 G needle and 12 cc syringe filled with RPMI supplemented with 10% heat-inactivated FBS and 2 mM EDTA. Single cell suspensions were filtered through 100 μm cell strainers. Red blood cells were lysed with ACK lysing buffer (Thermo Scientific) and resuspended in PBS with 1% heat-inactivated FBS and 2 mM EDTA. Cells were stained with the viability dye LIVE/DEAD Fixable Aqua Dead Cell Stain (Invitrogen) or Fixable Viability Dye eFluor 780 from eBioscience (San Diego, CA) and fluorochrome-conjugated monoclonal antibodies. Please see Supplementary Information for a list of antibody clones used. The Alexa Fluor 488 Protein Labeling Kit was used to fluorescently label Pfs25 for quantification of Pfs25-specific PCs by flow cytometry. Intracellular staining using the Foxp3/Transcription Factor Staining Buffer Set (eBioscience) was performed to detect IgG and Pfs25. Flow cytometric data were collected on a Fortessa flow cytometer (Becton Dickinson).

### Immunofluorescence and Confocal Microscopy

Tissue processing and confocal microscopy was performed as previously described^50^. Briefly, popliteal LNs were harvested, fixed with PLP buffer for 12 hours (0.05 M phosphate buffer containing 0.1 M L-lysine [pH 7.4], 2 mg/ml NaIO_4_, and 10 mg/ml paraformaldehyde), incubated in 30% sucrose for 6 hours, and embedded in OCT compound (Tissue-Tek). 30 μm sections were cut on a CM3050S cryostat (Leica) and adhered to Super Frost Plus Gold slides (Electron Microscopy Services). Frozen sections were blocked for 1–2 hours in PBS containing 0.3% Triton X-100 (Sigma), 1% normal mouse serum, 1% bovine serum albumin, and 10% normal goat serum. Sections were stained with directly conjugated antibodies for a minimum of 5 hours at RT or 12 hours at 4 °C in a humidity chamber in the dark. Stained slides were mounted with Fluoromount G (eBioscience) and sealed with a glass coverslip. Each section was visually inspected by epifluorescence light microscopy and high resolution images of several representative sections from different popliteal LNs were acquired using a SP8 confocal microscope (Leica), equipped with objectives with either 40X magnification (NA 1.3) or 63X magnification (NA 1.4). Fluorophore emission was collected on separate detectors with sequential laser excitation used to minimize spectral spillover. To calculate the total number of GCs per dLN, whole dLNs were serially sectioned and GCs were scored for each section. GC area was calculated by using the surface creation module in Imaris (Bitplane). Graph plots depict the widest area for each GC present in the dLNs examined as previously described^51^.

### Membrane Feeding Assays (MFA)

The ability of immune sera to block transmission of parasites to mosquitoes was measured using the Membrane Feeding Assay (MFA). *P. falciparum* NF54 cultures containing stage V gametocytes were used as the source for infective parasites. Gametocyte cultures were diluted with O^+^ RBC and heat-inactivated O^+^ serum to achieve a 0.15% ± 0.05% stage V gametocyte concentration at 50% hematocrit in a volume of 100 μL. Sera from each experimental group were pooled from 10 mice to create one test sample. For 1:8 sera dilutions, 20 μL of test sera and 20 μL naïve sera were mixed with 20 μL of human AB^+^ sera. After mixing, cultures were immediately fed to 3 day-old *Anopheles stephensi* mosquitoes. Eight days later, mercurochrome-stained oocysts were counted on midguts of ≥22 dissected mosquitoes per condition. The Transmission Reducing Activity (TRA) was the percent decrease in mean oocyst load compared to the naïve control group; the Transmission Blocking Activity (TBA) was the percent decrease in infected mosquitos compared to the naïve control.

### Statistical Analysis

Statistical analysis was performed using Prism 6 (GraphPad). Differences between two groups were compared using a Mann-Whitney *U* test for non-normal distributions. One-way analysis of variance with Tukey post-test was used to compare differences between more than two groups. (ns = not significant, *P < 0.05, **P < 0.01, ***P < 0.001, ****P < 0.0001).

## Additional Information

**How to cite this article**: Radtke, A. J. *et al*. Adjuvant and carrier protein-dependent T-cell priming promotes a robust antibody response against the *Plasmodium falciparum* Pfs25 vaccine candidate. *Sci. Rep.* **7**, 40312; doi: 10.1038/srep40312 (2017).

**Publisher's note:** Springer Nature remains neutral with regard to jurisdictional claims in published maps and institutional affiliations.

## Supplementary Material

Supplementary Information

## Figures and Tables

**Table 1:** Adjuvant-dependent effect on antibody magnitude and durability.

Adjuvant	Day 42 GMT (95% CI)		Day 126 GMT (95% CI)		Day 245 GMT (95% CI)		Antibody Half-life (Days) Mean ± SEM		ASCs Mean ± SEM	
Saline	**A**	170 (−753–2,484)	No data		No data		No data		No data	
Alhydrogel	**B**	12,964 (7,480–19,828)	**F**	10,832 (6,524–19,317)	**J**	4,309 (–245–12,025)	**N**	83.20 ± 17.56	**R**	51.29 ± 5.30
GLA–LSQ	**C**	215,344 (116,529–344,931)	**G**	112,782 (81,772–170,943)	**K**	51,642 (37,037–68,597)	**O**	88.00 ± 10.92	**S**	174.30 ± 21.54
CpG in SE	**D**	240,581 (150,073–353,202)	**H**	148,935 (103,345–227,086)	**L**	26,803 (14,400–43,758)	**P**	56.40 ± 4.07	**T**	79.00 ± 7.23
CFA/IFA	**E**	36,946 (28,469–46,584)	**I**	27,772 (14,725–56,553)	**M**	13,016 (−4,132–40,895)	**Q**	103.50 ± 24.69	**U**	108.00 ± 42.67

Mice were immunized with 1 μg Pfs25-EPA formulated in various adjuvants on day 0 and day 28. Sera were collected at the indicated time points and anti-Pfs25 IgG titers were determined by ELISAs. Shown is the geometric mean titer (GMT) with 95% confidence interval (CI) from 5–10 mice per group at peak titer (day 42), mid-point (day 126), and termination of the study: day 210 (CpG in SE or CFA/IFA) or day 245 (Alhydrogel and GLA-LSQ). Antibody half-lives were calculated using mid-point and end-point titers as described in the methods. Anti-Pfs25 IgG ASC numbers per million cells were calculated by ELISpots on day 245 for the Alhydrogel and GLA-LSQ groups and day 210 for the CpG in SE and CFA/IFA groups. Data are from 1 experiment and are representative of 3 similar experiments. One-way ANOVAs with Tukey post-tests were used to compare differences between anti-Pfs25 IgG titers, antibody half-lives, and the number of ASCs/million bone marrow cells. Statistically significant differences are denoted by the following: A–C (Day 42 Pfs25-EPA Saline vs Day 42 Pfs25-EPA GLA-LSQ) = ****, A–D (****), B–C (****), B–D (****), C–E (***), D–E (****), F–G (***), F–H (****), G–I (**), H–I (****), J–K (***), J–L (*), K–L (*), K–M (**), R–S (****), S–T (*), no significant differences between antibody half-lives (groups N–Q). (*P < 0.05, **P < 0.01, ***P < 0.001, ****P < 0.0001).
