# Supplementary material for: Adjuvant and carrier protein-dependent T-cell priming promotes a robust antibody response against the Plasmodium falciparum Pfs25 vaccine candidate
Source: Sci Rep. 2017 Jan 16;7:40312. doi: 10.1038/srep40312 (PMC5238395; doi:10.1038/srep40312)
Supplement: Supplementary Information [file srep40312-s1.pdf]

Supplementary Information

Adjuvant and carrier protein-dependent T-cell priming promotes a robust antibody response against the *Plasmodium falciparum* Pfs25 vaccine candidate

Andrea J. Radtke[1,5], Charles F. Anderson[2,5*], Nicolas Riteau[3,5], Kelly Rausch[2], Puthupparampil Scaria[2], Emily R. Kelnhofer[2], Randall F. Howard[4], Alan Sher[3,6], Ronald N. Germain[1,6], Patrick Duffy[2,6]

[1]Laboratory of Systems Biology, NIAID/NIH

[2]Laboratory of Malaria Immunology and Vaccinology, NIAID/NIH

[3]Laboratory of Parasitic Diseases, NIAID/NIH

[4]Infectious Disease Research Institute, Seattle, WA

[5]A.J.R., C.F.A., and N.R. contributed equally to this work

[6]A.S, R.N.G, and P.D. contributed equally to this work

*Correspondence to cfanderson@niaid.nih.gov

Supplementary Figures and Tables

- Figure S1
- Figure S2
- Figure S3
- Table S1
- Table S2
- Table S3

Supplementary Methods

- Proteins and Conjugation
- Adjuvants
- ELISAs
- B Cell ELISpots
- Antibodies

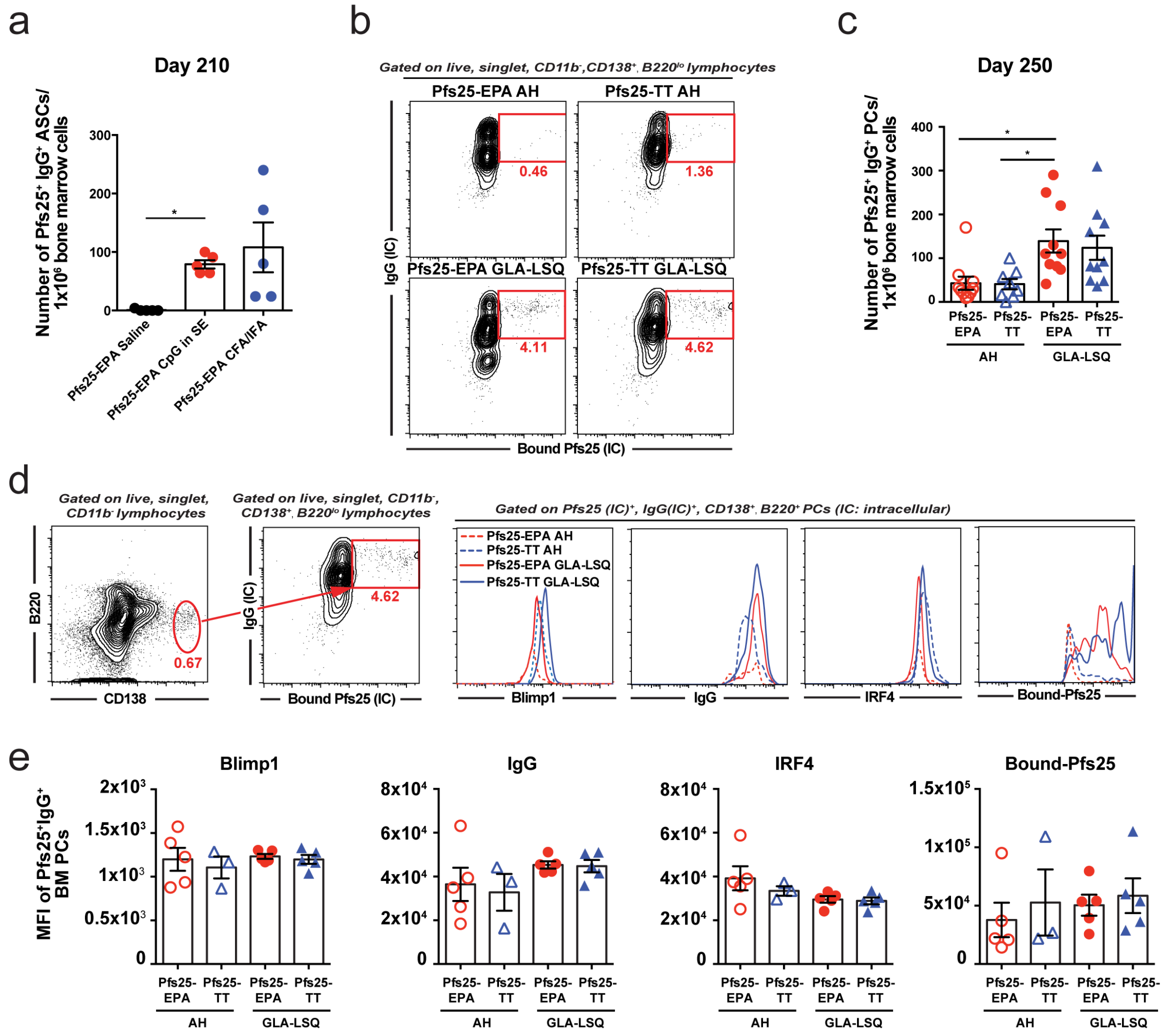

Figure S1. Effect of adjuvants and carrier proteins on anti-Pfs25 IgG responses

Mice were immunized i.m. with 1 µg Pfs25 alone or Pfs25-EPA formulated in various adjuvants on day 0 and day 28. Bone marrow was harvested at the indicated time points. **(a)** Number of ASCs specific for Pfs25 in the bone marrow of mice on day 210 calculated via ELISpot. Shown is the mean ± SEM, n=5 mice per group. **(b)** Expansion of $Pfs25^{+}IgG^{+}$ specific PCs in the bone marrow of immunized mice on day 250. IC denotes intracellular staining with Pfs25 and for IgG. **(c)** Number of $Pfs25^{+}IgG^{+}$ PCs in the bone marrow on day 250 calculated

via flow cytometry. Shown is the mean ± SEM, n=10 mice per group. **(d)** Representative flow plots and gating strategy used to examine protein expression by antigen-specific PCs in the bone marrow at day 250. **(e)** Graph plots of mean fluorescence intensities (MFI) of intracellular Blimp-1, IgG, IRF4, and bound Pfs25 in antigen-specific PCs from the bone marrow of mice immunized 250 days earlier. Data are from one experiment and are representative of 2 similar experiments. (* = $P < 0.05$; One-way ANOVAs with Tukey post-tests).

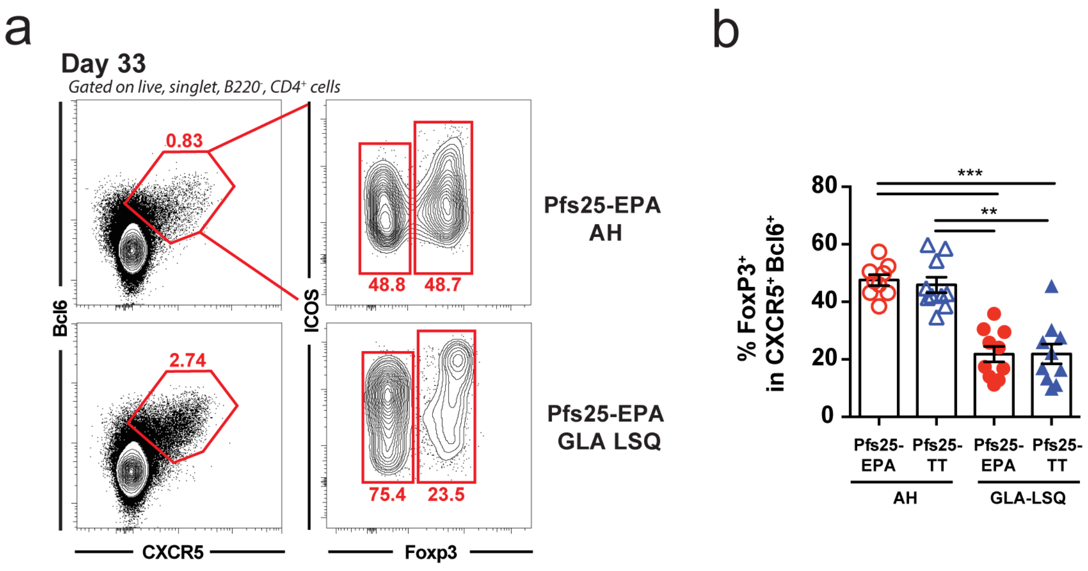

Figure S2. Evaluation of Tfh and Tfr cell responses at day 33

Mice were immunized i.m. with 1 μg Pfs25-EPA or Pfs25-TT in alhydrogel or GLA-LSQ on day 0 and day 28. dLNs were harvested on day 33 post-immunization and processed for flow cytometry. **(a)** Representative flow cytometry plots showing the frequency of $CXCR5^{+}Bcl6^{+}$ cells (left panels) and the frequency of $Foxp3^{-}$ (Tfh cells) or $Foxp3^{+}$ (Tfr cells) (right panels) within the $CD4^{+}$ T cell population after immunization with Pfs25-EPA in alhydrogel or GLA-LSQ. **(b)** Frequency of $Foxp3^{+}$ (Tfr cells) within the $CXCR5^{+}Bcl6^{+}CD4^{+}$ T cell population. Shown is the mean ± SEM, n=9-10 mice per group. Data are pooled from 2 similar experiments. (** = P < 0.01, *** = P < 0.001; One-way ANOVA with Tukey post-tests).

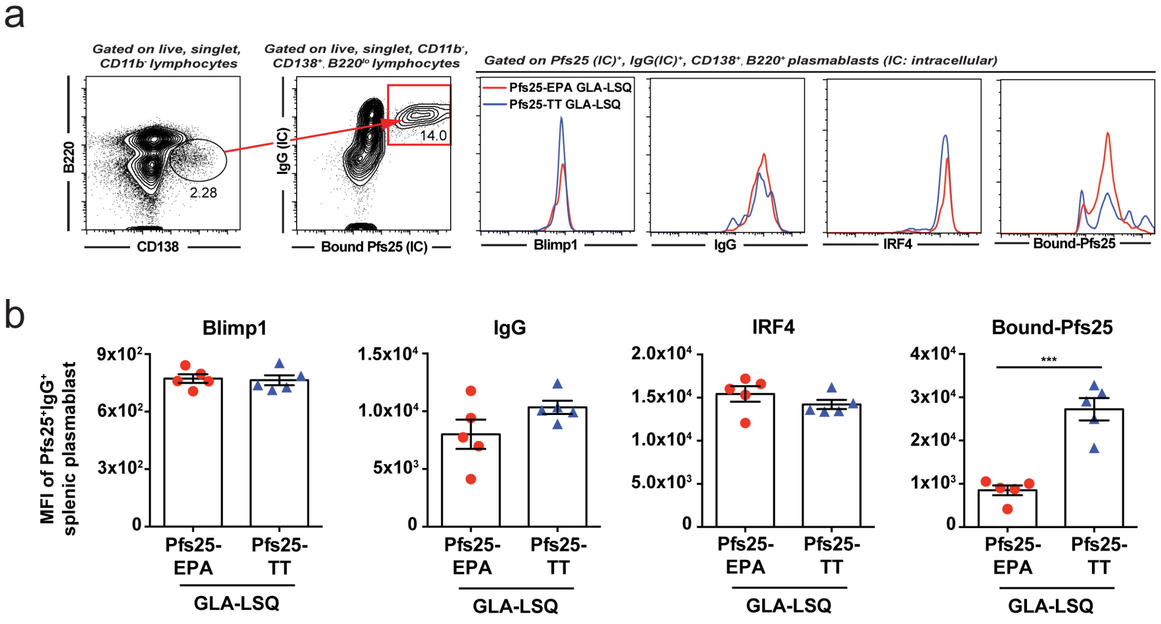

Figure S3. Flow cytometric assessment of splenic plasmablasts (Day 33)

Mice were immunized i.m. with 1 μg Pfs25-EPA or Pfs25-TT in alhydrogel or GLA-LSQ on day 0 and day 28. Spleens were harvested on day 33 post-immunization and processed for flow cytometry. **(a)** Representative flow plots and gating strategy used to examine protein expression by antigen-specific plasmablasts in the spleens at day 33. **(b)** Graph plots of mean fluorescence intensities (MFI) of intracellular Blimp-1, IgG, IRF4, and bound Pfs25 in antigen-specific plasmablasts from the spleens at day 33. Data are from one experiment and are representative of 2 similar experiments. Shown is the mean ± SEM, n=5 mice per group. (*** = P < 0.001; Mann-Whitney *U* test).

Adjuvant	Conjugate	Day 42 GMT (95% CI)	Day 255 GMT (95% CI)
Alhydrogel	Pfs25-Pfs25	**A** 4,055 (2,657-7,514)	**I** 1,117 (445-2,690)
	Pfs25-MSA	**B** 3,623 (1,528-9,226)	**J** 758 (73-2,665)
	Pfs25-EPA	**C** 8,060 (4,138-19,786)	**K** 2,115 (1,275-4,619)
	Pfs25-TT	**D** 9,238 (7,270-12,085)	**L** 1,577 (953-3,279)
GLA-LSQ	Pfs25-Pfs25	**E** 13,896 (9,975-30,678)	**M** 4,597 (2,703-10,880)
	Pfs25-MSA	**F** 18,068 (9,724-72,933)	**N** 5,780 (3,334-20,215)
	Pfs25-EPA	**G** 110,157 (81,018-170,691)	**O** 32,357 (22,438-51,901)
	Pfs25-TT	**H** 218,011 (161,480-304,411)	**P** 30,186 (27,573-33,190)

Axis (both columns, log scale): 100, 1000, 10000, 100000

Table S1. Evaluation of anti-Pfs25 antibody response following immunization with different conjugate vaccines

Mice were immunized i.m. with 1 µg of the indicated conjugate vaccines on day 0 and day 28. Sera were collected and anti-Pfs25 IgG titers were determined by ELISAs. Shown is the geometric mean titer (GMT) with 95% confidence interval (CI) from 5–10 mice per group at peak titer (day 42) and termination of the study (day 255). Data are from 1 experiment and are representative of 3 similar experiments. One-way ANOVAs with Tukey post-tests were used to compare differences between anti-Pfs25 IgG titers at day 42 (peak) and day 255 (end-point). Bolded letters (A-P) refer to data in that row, for example 'A' is the GMT (95% CI) for mice immunized with Pfs25-Pfs25 in Alhydrogel at day 42. Statistically significant differences are denoted by the following: A-G (Day 42 Pfs25-Pfs25 Alhydrogel vs Day 42 Pfs25-EPA GLA-LSQ) = ****. A-H (****), B-G (****), B-H (****), C-G (****), C-H (****), D-G (****), D-H (****), E-G (****), E-H (****), F-G (**), F-H (****), G-H (****), I-O (****), I-P (****), J-O (****), J-P (****), K-O (****), K-P (****), L-O (****), L-P (****), M-O (****), M-P

(****), N-O (****), N-P (***), G-O (****), and H-P (****). Colored bars are a graphical representation of the GMT for all groups. (*** = $P < 0.001$, **** = $P < 0.0001$).

Antibody Response	Conjugate	Adjuvant	Day 42 GMT (95% CI)	Day 126 GMT (95% CI)	Day 236 GMT (95% CI)	Antibody Half-life (Days) Mean ± SEM
anti-TT	Pfs25-TT	Alhydrogel	**A** 4,046 (2,495-6,561)	**C** 2,344 (1,233-5,260)	**E** 916 (416-2,018)	**G** 88.99 ± 9.52
	Pfs25-TT	GLA-LSQ	**B** 53,827 (41,400-69,984)	**D** 33,963 (24,322-47,424)	**F** 16,596 (10,447-26,363)	**H** 101.90 ± 8.31
	Pfs25-EPA	Alhydrogel	<32	No data	No data	No data
	Pfs25-EPA	GLA-LSQ	<32	No data	No data	No data
anti-EPA	Pfs25-EPA	Alhydrogel	**I** 12,133 (6,792-21,677)	**K** 8,241 (4,315-15,739)	**M** 2,472 (971-6,295)	**O** 71.67 ± 10.37
	Pfs25-EPA	GLA-LSQ	**J** 74,817 (46,773-119,674)	**L** 34,434 (20,323-58,345)	**N** 15,136 (10,116-22,646)	**P** 88.22 ± 6.25
	Pfs25-TT	Alhydrogel	<24	No data	No data	No data
	Pfs25-TT	GLA-LSQ	<24	No data	No data	No data
			100 1000 10000 100000	100 1000 10000 100000	100 1000 10000 100000	0 100 200 300 400 500

Table S2. Humoral immune response elicited by carrier proteins

Mice were immunized i.m. with 1 μg of Pfs25-EPA or Pfs25-TT on day 0 and day 28. Anti-TT and anti-EPA IgG titers were determined by ELISAs from sera collected at the indicated time points. Shown is the geometric mean titer (GMT) with 95% confidence interval (CI) from 10 mice per group at peak titer (day 42), mid-point (day 126), and termination of the study (day 236). Data are from 1 experiment and are representative of 3 similar experiments. Bolded letters (A-P) refer to data in that row, for example ‘A’ is the peak (day 42) anti-TT IgG GMT (95% CI) for mice immunized with Pfs25-TT in alhydrogel. One-way ANOVAs with Tukey post-tests were used to compare differences between peak (day 42), mid-point (day 126), and end-point titers (day 236) for anti-EPA or anti-TT separately, e.g. rows A-F or I-N. Statistically significant differences are denoted by the following: A-B (Day 42 anti-TT IgG titers from mice immunized with Pfs25-TT in Alhydrogel vs Day 42 anti-TT IgG titers from mice immunized with Pfs25-TT GLA-LSQ) = ****. C-D (****), E-F (**), B-F (****), I-J (****), K-L (*),

and J-N (****). (* = P < 0.05, ** = P < 0.01, **** = P < 0.0001). Antibody half-lives were calculated with titer data from day 126 and day 236 and differences between groups (G and H) and (O and P) were compared with a Mann-Whitney *U* test. G-H = not significant (ns), O-P = ns. Colored bars are a graphical representation of the GMT (A-F and I-N) or mean (G, H, O, and P). Negative controls include sera collected from mice immunized with the irrelevant conjugate vaccine. For example, mean anti-TT IgG titers for mice immunized with Pfs25-EPA in alhydrogel or GLA-LSQ were below the detection limit at day 42 (<32) and mean anti-EPA IgG titers for mice immunized with Pfs25-TT in alhydrogel or GLA-LSQ were below the detection limit at day 42 (<24).

	Nonamer	Starting position	Score
Pfs25	VCSCNIGKV	110	3.20
EPA	YIAGDPALA	473	6.03
	YRTSLTLAA	515	4.55
	LAAPEAAGE	521	4.05
	YLAARLSWN	301	3.18
	LTCPVAAGE	373	3.05
TT	YIGPALNIV	645	6.53
	YLIPVASSS	1129	5.90
	YSDPVNNDT	10	7.77
	YFPSVISKV	595	5.47
	YNAPGIPLY	1229	4.78
	FSTPIPFSY	855	4.04
	FNAYLANKW	1023	3.98
	IFGPGPVLN	159	3.88
	VDDALINST	582	3.63
	YNDMFNNFT	943	3.51
	YGFTEIELG	356	3.33
	YVPTFDNVI	202	3.30
	FGGQDANLI	275	3.27
	FNPPSSLIE	59	3.21
	YKSNAASTI	542	3.16
	YYDPNYLRT	72	3.15
	YSGPDKEQI	763	3.03

(Score > 3.0 = Good I-A^b binding candidate)

Table S3. Identification of putative $CD4^+$ T cell epitopes in carrier proteins

Pfs25 (18 kDa), EPA (67 kDa) and TT (151 kDa) protein sequences were submitted to an algorithm developed by Marc Jenkins' laboratory designed to predict I-A^b binding epitopes[1]. This algorithm is based on the fact that peptides bind the I-A^b molecule via a 9 amino acid core sequence, a nonamer. Furthermore, peptide positions 2, 5, 7 and 8 are the main TCR contacts for I-A^b binding. Nonamers with a score >3 were considered good I-A^b binding

candidates and listed in the table together with the starting position in their respective protein sequences.

Supplementary Methods

Proteins and Conjugation

Recombinant Pfs25 (average MW=18 kDa) from *P. falciparum* NF54 was produced in *Pichia pastoris*. Exoprotein A (EPA) (average MW=67 kDa) is a non-toxic mutant of exotoxin A from *Pseudomonas aeruginosa* produced in *E. coli*. Tetanus toxoid was provided by Novartis Vaccines and Diagnostics. Mouse serum albumin was purchased from Sigma Aldrich (essentially globulin free, ≥99% by agarose gel electrophoresis). Antigen conjugates were manufactured at the LMIV based on a published method[2] using thioether crosslinking chemistry. In summary, for each conjugate, Pfs25 was thiolated by treatment with S-acetylthioglycolic acid N-hydroxysuccinimidyl ester (SATA) and deacetylation with hydroxylamine. The carrier was activated with EMCS (6-Maleimidohexanoic acid N-hydroxysuccinimide ester (EMCS), and the two components were mixed to form the nanoparticle. Conjugates were purified and fractionated by Size Exclusion Chromatography (SEC) and unconjugated monomers were removed. Conjugate fractions were collected after discarding 10% of the total area under the conjugate peak (5% each from the leading and tailing ends of the conjugate peak) to remove high and low molecular weight conjugates. Average molecular mass and hydrodynamic radius of the conjugates were determined by size exclusion chromatography coupled with light scattering (SEC-MALS). Protein

composition was determined by least squares regression following amino acid analyses. The weighted average molar mass of Pfs25-EPA was 823 kDa with a 90% MW distribution of 170-2,558 and hydrodynamic radius (Rh, determined by Dynamic Light Scattering) of 15.4 (± 0.4) nm. Weighted average MW of Pfs25-TT was 2,473 kDa, with a 90% MW distribution of 510-6844 and an Rh of 9.4 (± 0.3) nm. Pfs25-MSA had a weighted average MW of 1,035 kDa, with a 90% MW distribution of 217-2,936 and an Rh of 17.3 (± 0.6) nm. Pfs25-Pfs25 had a weighted average MW of 713 kDa, with a 90% MW distribution of 174-2,058 and an Rh of 16.0 (± 0.9) nm. Protein composition (antigen/carrier ratio) of the conjugates were determined by least squares regression following amino acid analyses.

Adjuvants

GLA-LSQ and CpG in SE were obtained from the Infectious Disease Research Institute, Seattle, WA. GLA-LSQ is a liposome-based adjuvant containing GLA, a synthetic TLR4 agonist, and the saponin QS21. QS21 is a saponin derived from the bark of *Q. saponaria*. GLA/QS21 content in GLA-LSQ formulations was 0.1 mg/mL GLA, 0.2 mg/mL QS21 and 2 mg/mL phospholipid in 0.05 mL per dose, or 5 µg GLA/10 ug QS21/100 µg phospholipid per dose. CpG 1826 was formulated in an oil-in-water stable emulsion. The CpG 1826 concentration in CpG-SE was 0.4 mg/mL CpG/2% SE in a final volume of 0.05 mL per dose, or 20 µg CpG in 2% SE per dose. Complete and incomplete Freund's adjuvants were purchased from Sigma Aldrich. Alhydrogel (2%

aluminum hydroxide gel) was produced by Brenntag Biosector, Denmark. Alhydrogel was 1.6 mg/mL per 0.05 mL dose, or 80 μg Alhydrogel per dose.

ELISAs

Immulon 4 HBX flat bottom microtiter plates (Dynex Technologies) ELISA plates were coated with 1 μg/ml of antigen in a volume of 100 μL per well in carbonate coating buffer (pH 9.6) overnight at 4°C. After blocking in 5% skim milk in TBS blocking buffer in a volume of 320 μL per well for 2 hrs, samples were serially diluted in TBS/5% milk and plated in triplicate in a volume of 100 μL per well and incubated at room temperature for 2 hours. Plates were washed 4 times and alkaline phosphatase labeled goat anti-mouse IgG (H+L) (Kirkegaard & Perry Labs, Inc) was added at a dilution of 1:1,000 in a volume of 100 μL per well and incubated at room temperature for 2 hours. After washing 4 times, dissolved phosphatase substrate tablets (Sigma) were added in a volume of 100 μL per well and plates were incubated for 20 minutes before optical densities (OD) were measured with a Spectramax 340PC (Molecular Devices). Each ELISA plate contained an internal serum standard from which a four-parameter curve was calculated with Softmax software. ELISA Units were assigned to test samples based on the sera dilution that gave an OD of 1.0, adjusted to the internal standard.

B Cell ELISpots

ELISpots were performed using the B cell ELISpot kits from Mabtech. Multiscreen-IP PVDF 96-well filter plates (type MAIPSWU) were treated with 70% ethanol prior to coating with antigen at a concentration of 10 μg/ml in a volume of 100 μL per well and incubated overnight at 4°C. The next day plates were washed and blocked in a volume of 250 μL per well in Iscove's Modified Dulbecco's Medium (IMDM) containing 10% FBS at room temperature. Bone marrow suspensions were flushed from the femors and tibias of mice using 30G needles with HBSS. After treatment with ACK lysing buffer for 5 minutes, cells were suspended in IMDM/10% FBS in volumes to be serially plated at 250,000, 125,000, 62,500, and 31,250 cells per well in a volume of 100 μL per well in triplicate and incubated at 37°C for approximately 16 hours. After washing, anti-IgG-biotin detection antibody was added at a concentration of 1 μg/ml in a volume of 100 μL per well and incubated at room temperature for 2 hours. After washing again, streptavidin-AP was diluted to 1:1,000 in PBS and added in a volume of 100 μL per well and plates were incubated at room temperature for 1 hour. After final washing, substrate solution was added and spots were allowed to develop for 20 minutes. The reaction was stopped by washing with water and once plates were dry images were captured using a CTL ImmunoSpot analyzer (Cellular Technology Limited, Shaker Heights, OH). Images were then manually inspected and counted. Images with clear resolution of spots (usually >10 and <40 spots per well) were used for calculating the number of spot forming units per million cells.

Antibodies

All of the following antibodies were purchased from eBioscience (San Diego, CA), BioLegend (San Diego, CA), or BD Biosciences (San Jose, CA): anti-B220 (clone RA3-682), anti-CD138 (281-2), anti-IgD (11-26c.2a), anti-IgG (Poly4053), anti-GL7 (GL7), anti-CD11b (M1/70), anti-CD4 (GK1.5), anti-CD44 (IM7), anti-CXCR5 (SPRCL5), anti-PD-1 (29F.1A12), anti-ICOS (7E.17G9), anti-Foxp3 (FJK-16s), and anti-Bcl6 (K112-91). Unconjugated primary antibodies were stained with Alexa Fluor-conjugated secondary antibodies (Invitrogen).

References

1 Nelson, R. W. *et al.* T cell receptor cross-reactivity between similar foreign and self peptides influences naive cell population size and autoimmunity. *Immunity* **42**, 95-107, doi:10.1016/j.immuni.2014.12.022 (2015).

2 Jones, D. S. *et al.* A Method for Producing Protein Nanoparticles with Applications in Vaccines. *PLoS One* **11**, e0138761, doi:10.1371/journal.pone.0138761 (2016).
